# Temporal Transcriptomic Changes in the Cingulate Cortex of Neuropathic Pain Mice

**DOI:** 10.3390/biomedicines14071495

**Published:** 2026-07-01

**Authors:** Guo-Quan Yao, Zhen-Ru Yuan, Xin-Tong Qiu, Cheng-Guo Jiang, Chong Zhang, Si-Zhe Feng, Guang-Xi Piao, Hong Ma, Zi-He Zhu, Yu-Gang Diao, Felipe Fregni, Yang Bai

**Affiliations:** 1 Department of Anesthesiology, General Hospital of Northern Theater Command, Shenyang 110016, China; yaoguoquan1983@163.com (G.-Q.Y.); 13591690202@163.com (Z.-R.Y.); zihezhuu@163.com (Z.-H.Z.); 2 Key Laboratory of Perioperative Critical Medicine of Liaoning Province, Shenyang 110016, China; 3 Shenyang Clinical Medical Research Center for Anesthesiology & Perioperative Medicine, Shenyang 110016, China; 4 Department of Anatomy, Histology and Embryology, Preclinical School of Medicine, Air Force Medical University, Xi’an 710032, China; xintongqiu@fmmu.edu.cn; 5 Department of Neurosurgery, General Hospital of Northern Theater Command, Shenyang 110016, China; 19802466466@163.com (C.-G.J.); aiqingstupid@aliyun.com (C.Z.); fengsizhe@sohu.com (S.-Z.F.); 6 Department of Anesthesiology, Jilin Provincial People’s Hospital, Changchun 130021, China; 13331544443@163.com; 7 Department of Anesthesiology, First Affiliated Hospital, China Medical University, Shenyang 110001, China; mahong5466@163.com; 8 Neuromodulation Center and Center for Clinical Research Learning, Spaulding Rehabilitation Hospital and Massachusetts General Hospital, Harvard Medical School, Boston, MA 02115, USA

**Keywords:** neuropathic pain, cingulate cortex, transcriptomics, synaptic plasticity, neuroinflammation

## Abstract

**Background**: Neuropathic pain (NP), a debilitating condition resulting from nervous system lesions, is poorly managed by current therapies. The cingulate cortex is crucial for affective pain processing, yet a comprehensive spatiotemporal understanding of its molecular changes in NP is lacking. **Methods**: We performed RNA sequencing to profile transcriptomic alterations in the anterior cingulate (ACC) and midcingulate (MCC) cortices of mice at two and four weeks after spared nerve injury. Bioinformatics analyses, including differential expression, functional enrichment, weighted gene co-expression network analysis, and protein–protein interaction (PPI) network construction, were employed. **Results**: We identified widespread, time-dependent transcriptional dysregulation in both regions, with differentially expressed genes increasing over time. Functional analyses confirmed central roles for synaptic plasticity and neuroinflammatory pathways, and further identified pathways related with neurodegeneration and mitochondrial dysregulationin NP pathogenesis. Subregion analysis revealed that the ACC exhibited broader pathway alterations than the MCC, including neuroinflammation (early phase) and mitochondrial dysfunction/neurodegeneration (late phase), indicating a progressive stress response unique to the ACC. PPI analysis identified stage-specific hub genes (e.g., early interferon-stimulated genes and late ribosomal proteins in ACC; persistent extracellular matrix components in MCC). **Conclusions**: This study provides a detailed transcriptomic atlas of the cingulate cortex in NP, reinforcing known synaptic and neuroinflammatory mechanisms, and suggests a possible role of mitochondrial dysregulation in NP pathogenesis. The findings provide a basis for further mechanistic studies.

## 1. Introduction

Neuropathic pain (NP), a heterogeneous condition affecting roughly 10% of individuals worldwide, is defined by a lesion or disease of the somatosensory nervous system [1]. This chronic pain state represents a persistent therapeutic challenge in pain medicine [2]. First-line pharmacotherapies often reach a therapeutic ceiling, constrained by modest efficacy and dose-limiting adverse effects [3]. This unresolved clinical predicament underscores the imperative to move beyond symptomatic management and decipher the intricate pathophysiology driving NP. A pivotal step in this direction is the comprehensive molecular profiling of pain matrix, which is expected to reveal novel mechanistic insights and unlock new, targeted therapeutic strategies [4,5].

The cingulate gyrus is a pivotal cortical hub for processing pain and emotion. It comprises four subregions—the anterior (ACC), midcingulate (MCC), posterior, and retrosplenial cortices—each defined by distinct cytoarchitecture, connectivity, and functional profiles. Among these, the ACC and MCC have been most extensively studied in the context of pain and are believed to mediate the affective dimension of pain [6,7]. Early behavioral evidence from rodent studies indicated that activation of ACC promotes behavioral sensitization and pain-related aversion [8,9,10,11]. Subsequently, the widespread application of optogenetics has further elucidated the modulatory roles of distinct cingulate neuronal subtypes [12,13] and their fiber connections in pain regulation [14,15,16,17,18]. Cortical long-term potentiation (LTP) in the ACC serves as a cellular model of pathological pain [19]. Nerve injury triggers lasting potentiation at excitatory synapses, involving enhanced presynaptic glutamate release and postsynaptic glutamatergic receptor recruitment [20]. Recently, transcriptomic approaches have delineated ACC-wide gene expression changes in chronic pain states, confirming synaptic plasticity pathways and uncovering new roles of neuroinflammatory and apoptotic processes, thereby deepening our molecular understanding of pain pathogenesis [21,22,23,24,25].

The MCC is implicated in attention, cognition, emotion, and sensory processing, and its dysfunction is associated with various neurological and psychiatric disorders [26]. Human neuroimaging studies have long identified the MCC as a key hub for the perception of acute nociceptive pain [27]. In rodent studies, however, the MCC was historically considered a caudal extension of the ACC and was only later recognized as a distinct cingulate subregion following the work of Vogt et al. [6]. While pain research has predominantly focused on the ACC [7], recent investigations have begun to elucidate the specific contribution of the MCC to pain modulation. Emerging evidence indicates that the MCC contributes to both sensory and affective dimensions of pain [28,29] via different neural circuits [30,31,32]. Despite these advances focusing on cellular mechanisms, remarkably little is known about the molecular basis underlying the neurobiology of the MCC in chronic pain.

As noted above, although a series of studies have uncovered pain-related functional adaptations in the cingulate cortex via RNA sequencing [21,22,23,24,25], these investigations focused exclusively on the ACC and only examined early transcriptomic changes within two weeks post-injury. We hypothesized that the ACC and MCC exhibit distinct transcriptomic signatures following NP, owing to their different neuroanatomical connectivity and functional profiles in pain processing. Moreover, the transcriptional landscape of each subregion undergoes dynamic evolution after nerve injury. Therefore, we systematically characterized the transcriptomic landscape of the cingulate cortex focusing on its temporal (two and four weeks post-modeling) and spatial (ACC and MCC subregions) dynamics in a mouse NP model induced by spared nerve injury (SNI). This approach elucidates the complex pathological reorganization within the cingulate cortex, providing an integrated perspective on its role in NP modulation.

## 2. Materials and Methods

### 2.1. Animals

All animal experiments were approved by the Ethics Committee of the General Hospital of Northern Theater Command and were conducted in accordance with the National Institutes of Health Guide for the Care and Use of Laboratory Animals. Eighty-five adult male C57BL/6 mice (8 weeks old; 20–25 g) were obtained from the hospital’s experimental animal department. Mice were maintained under a reversed 12 h/12 h dark/light cycle with ad libitum access to food and water.

### 2.2. Neuropathic Pain Model Induction and Behavioral Validation

The SNI model was selected for three main reasons. First, it produces robust, long-lasting mechanical hypersensitivity, making it suitable for chronic pain studies of up to four weeks. Second, it offers a highly reproducible lesion pattern with a simple and stable preparation [33]. Third, given that two of three published cingulate transcriptomic studies on NP have used this model [21,23,25], its adoption ensures comparability with previous work. The mice were deeply anesthetized and a skin incision was made in the left hind leg to expose the biceps femoris muscle. The underlying sciatic nerve and its three terminal branches—the sural, common peroneal, and tibial nerves—were then carefully isolated. Both the common peroneal and tibial nerves were tightly ligated with 6-0 silk suture and transected distal to the ligature, leaving the sural nerve intact. The muscle and skin were subsequently sutured. Sham-operated animals underwent the same surgical exposure of the sciatic nerve without ligation or transection.

Animals were randomly assigned to the sham group and the NP model group using a computer-generated random number sequence (Microsoft Excel, RAND function). To minimize potential confounders, all behavioral tests were conducted at the same time of day (9:00–12:00) to control for circadian effects. The order of testing and treatment was balanced across groups. Cage locations were randomized on the rack to avoid position effects. The experimenters performing behavioral assessments and data analysis were blinded to group allocation.

Paw mechanical withdrawal thresholds were determined using the ascending stimulus method, as described in previous studies [34,35]. Prior to baseline assessments, mice were acclimated to the testing environment for three consecutive days. On the test day, each animal was placed inside an inverted transparent plastic box on a raised metal mesh floor and allowed to acclimate for 30 min before measurement. A set of ten calibrated von Frey hairs (Stoelting, Kiel, WI, USA) with logarithmically incrementing bending forces (0.008, 0.02, 0.04, 0.07, 0.16, 0.4, 0.6, 1.0, 1.4, and 2.0 g; corresponding to 0.078, 0.196, 0.392, 0.784, 1.568, 3.92, 5.88, 9.8, 13.72, and 19.6 mN) was applied to the plantar surface of the hind paw in ascending order of force until a withdrawal response was elicited. Positive responses were defined as rapid paw withdrawal, licking, shaking of the paw, or vocalization. Each filament was applied five times to the same testing area, with a duration of three seconds per application. The minimal bending force that induced a paw withdrawal reflex in at least three out of five applications was recorded as the threshold.

### 2.3. Study Design and Tissue Harvesting

**Figure A1:**
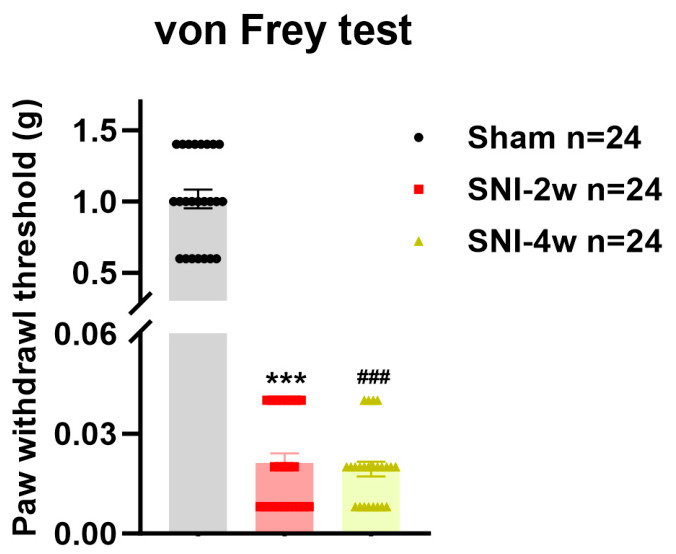
Behavioral tests. Compared to sham group, SNI mice exhibited markedly decreased paw withdrawal thresholds (one-way ANOVA: *** *p* < 0.001 for SNI 2w; ^###^ *p* < 0.001 for SNI 4w, both vs. sham).

Prior to SNI model induction, all animals were screened for baseline mechanical sensitivity of the left plantar surface using von Frey filaments. Mice exhibiting normal withdrawal thresholds (0.6, 1.0, or 1.4 g) were included, while four animals with abnormal thresholds (<0.6 g or >1.4 g) were excluded. The remaining 81 mice were randomly assigned to three groups: sham, SNI-2w, and SNI-4w. The SNI procedure was performed on the left side to induce NP. Mechanical thresholds of the ipsilateral plantar surface were reassessed at two and four weeks post-surgery to confirm successful model establishment. Of the 54 SNI-operated animals, 52 met the success criterion (withdrawal threshold ≤0.04 g), yielding a model success rate of 96.3%. One day after the final behavioral test, mice were euthanized by rapid cervical dislocation. From each group, 24 animals were selected for cingulate cortex collection. The behavioral results are shown in Figure A1.

The cingulate cortex lies adjacent to the medial frontal cortex, regions previously shown to differentially modulate pain [9,36]. To ensure precise sampling, 300 μm coronal brain sections containing the bilateral cingulate cortex were prepared in ice-cold artificial cerebrospinal fluid (in mM: 2.6 KCl, 124 NaCl, 1 MgCl_2_, 0.5 CaCl_2_, 1.23 NaH_2_PO_4_, 26.2 NaHCO_3_, 5 kynurenic acid, 212.7 sucrose, 10 dextrose, pH 7.4) using a vibratome, as described in prior patch-clamp studies [37,38]. Target tissues were micro-punched with a 15-gauge needle under a microscope based on the coordinates from Paxinos and Franklin’s *The Mouse Brain in Stereotaxic Coordinates*, 4th edition, and then snap-frozen on dry ice.

**Figure 1:**
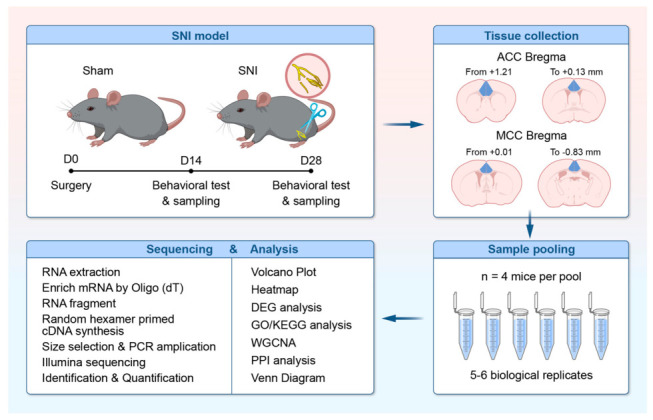
Systematic workflow of transcriptome analysis in the cingulate cortex of neuropathic pain mouse model.

Total RNA was extracted separately from the ACC (bregma +1.21 to +0.13 mm) and MCC (bregma +0.01 to –0.83 mm) (Figure 1). For each region and experimental group, six independent biological replicate RNA pools were prepared. Each pool consisted of bilateral cingulate tissue combined from four animals (Figure 1), yielding approximately 30 mg tissue per biological replicate (each animal contributed about 7–9 mg of ACC and MCC tissue), which was sufficient for subsequent RNA sequencing. The sample size of *n* = 6 per group was chosen based on our previous experience in pain-related transcriptomics [23]. While most current transcriptomic studies of the cingulate cortex in pain use only *n* = 3 [21,22,24,25,39], a larger sample size can theoretically minimize biological variance, enable reliable dispersion estimation, and improve the detection of differentially expressed genes (DEGs).

To minimize RNA degradation, all tools and surfaces were treated overnight with 0.1% diethylpyrocarbonate in 0.01 M phosphate-buffered saline before use. All tissue samples were promptly stored at –80 °C. During subsequent transcriptomic processing, one ACC sample from the SNI-2w group was excluded due to suboptimal storage conditions. The corresponding MCC sample (from the same animal pool) was also removed to maintain consistency in sample pairing across groups. Thus, the SNI-2w group had five biological replicates per region, whereas other groups had six.

### 2.4. RNA Sequencing

Total RNA was extracted from tissue samples with the TRIzol reagent (Thermo Fisher, Carlsbad, CA, USA). The RNA quality was verified on an Agilent 5300 Bioanalyzer and quantified using a NanoDrop 2000 (Thermo Fisher). The construction of the sequencing library required RNA samples to meet the following quality thresholds: a total amount of 1 μg, a concentration of ≥30 ng/μL, an RNA Integrity Number (RIN) > 6.5, and an OD260/280 ratio between 1.8 and 2.2. mRNA was enriched from total RNA using Dynabeads Oligo (dT) (Thermo Fisher) and fragmented via incubation with divalent cations (Magnesium RNA Fragmentation Module, NEB, Ipswich, MA, USA). The resulting fragments were reverse-transcribed into cDNA using SuperScript™ II Reverse Transcriptase (Invitrogen, Carlsbad, CA, USA). Second-strand cDNA synthesis was subsequently performed in the presence of dUTP to generate U-labeled DNA, utilizing a cocktail of E. coli DNA polymerase I, RNase H, and dUTP Solution (all from Thermo Fisher and NEB). Following end-repair and A-tailing, the products were ligated to dual-indexed adapters. Adapter-ligated fragments were size-selected using AMPureXP beads. The libraries were then treated with the heat-labile UDG enzyme (NEB) to digest the U-labeled strand prior to PCR amplification. The PCR was carried out under the following conditions: 95 °C for 3 min; 8 cycles of 98 °C for 15 s, 60 °C for 15 s, and 72 °C for 30 s; with a final extension at 72 °C for 5 min. The resulting cDNA libraries, with an average insert size of 300 ± 50 bp, were sequenced on an Illumina NovaSeq X Plus platform for 2 × 150 bp paired-end reads.

### 2.5. Transcriptomic Analysis

RNA sequencing yielded 845.17 million paired-end reads (2 × 150 bp). Raw reads were processed with fastp (v0.22.0) to remove adapter sequences and low-quality bases, followed by quality assessment using FastQC (v0.11.9). After quality control, 252.10 Gb of clean reads were retained and aligned to the mouse reference genome using HISAT2 (v 2.2.1). Gene-level read counts were obtained using featureCounts (v2.0.1). For differential expression analysis, the raw read counts were directly used as input for DESeq2 (v1.48.1). Raw counts were also transformed to log2 counts per million [log_2_(CPM + 1)] using the cpm function in the edgeR package (v4.4.0) for other downstream analyses. Principal component analysis (PCA) was performed on the scaled expression matrix using the PCA function from the FactoMineR package (v2.11) in R. The first two principal components were visualized with the fviz_pca_ind function from the factoextra package (v1.0.7), with samples colored according to experimental group. Differential expression analysis was performed with DESeq2, applying thresholds of |fold change (FC)| > 1.5 and adjust p-value (padj) < 0.05 to define DEGs. The choice of a moderate |log_2_FC| > 0.58 threshold was based on a review of rodent transcriptomic studies on pain-related cortical plasticity in the ACC, where reported FC values typically fell between 1 and 2 [21,22,23,24,25,39]. When combined with a strict padj < 0.05 correction, this cutoff provides a practical balance between capturing authentic biological signals and minimizing false-positive findings.

Venn diagrams were generated using ggVennDiagram (version 1.5.3) in R/Bioconductor. Functional enrichment analysis of DEGs for Gene Ontology (GO) terms and Kyoto Encyclopedia of Genes and Genomes (KEGG) pathways was carried out using the clusterProfiler R package (v4.16.0).

To construct the co-expression network, we employed the Weighted Gene Co-expression Network Analysis (WGCNA) package (version 1.73) to calculate similarity between gene expression profiles [40]. A scale-free network was built using the WGCNA method. The adjacency matrix was converted into a topological overlap matrix through hierarchical clustering, and different modules were identified by applying a dynamic tree-cut algorithm. Parameter settings were as follows: minModuleSize = 50, pamRespectsDendro = FALSE, cutHeight = 0.3. The soft-thresholding power β was selected from 1 to 20 based on the scale-free topology fit index (signed R^2^). The optimal β was defined as the smallest power achieving signed R^2^ ≥ 0.8, a widely accepted criterion that ensures a reasonable approximation to a scale-free topology while avoiding excessive network sparseness [40,41], resulting in β = 3 for ACC and β = 14 for MCC.

To investigate functional connectivity among DEGs, protein–protein interactions between significant DEGs across cingulate subregions and time points were predicted using the STRING database (v12.0) [42], with the minimum required interaction score set to “high confidence” (0.7) and disconnected nodes hidden from the network visualization. The cytoHubba’s Maximal Clique Centrality score was used to identify top hub DEGs and their sub-networks. The data were visualized using Cytoscape (v3.7.0) [21].

## 3. Results

### 3.1. Overview of RNA-Seq Data and Basic Alignment Metrics

**Figure A2:**
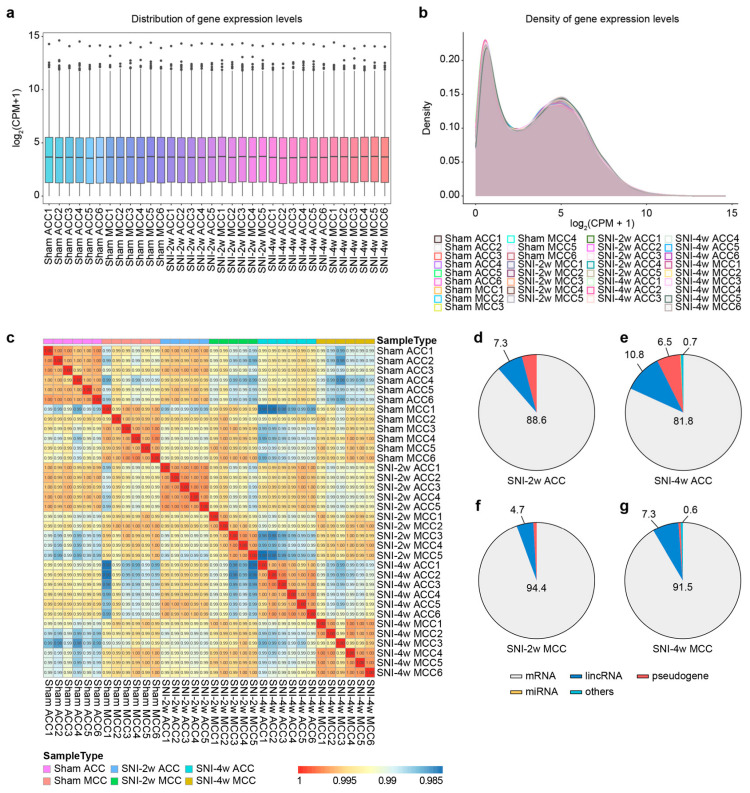
The quality of RNA-seq data from each sample. (**a**) Boxplot showing the distribution of gene expression levels in each cingulate cortex sample. (**b**) Distribution diagram showing the density of gene expression levels in each cingulate cortex sample. (**c**) Pearson correlation analysis results. (**d**–**g**) The constituent ratio of the identified DEGs.

Transcriptome profiling of cingulate cortex subregions was performed at 14 and 28 days following SNI or sham surgery (Figure 1). After quality control, an average of 24,659,123 clean reads per sample were obtained from approximately 25 million raw reads and aligned to the reference genome with a mapping rate of 98.5–99.0% (Table A1). Detected genes were quantified and their distribution across samples is summarized in Figure A2a,b. To assess experimental consistency and sample suitability, we evaluated inter-sample correlations based on normalized expression values, as visualized in the correlation heatmap (Figure A2c).

### 3.2. Altered Transcriptional Signatures in the Cingulate Cortex After SNI

**Figure 2:**
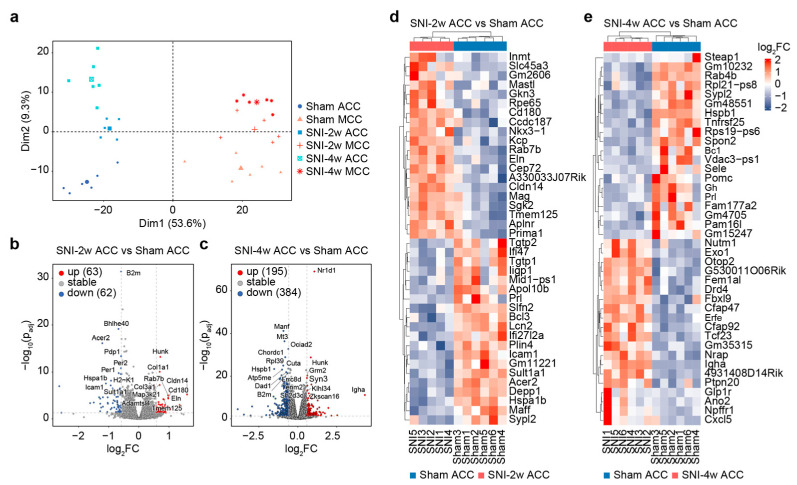
General transcriptomic outcomes of ACC. (**a**) Principal component analysis indicated a near complete separation of genes among these six groups. (**b**,**c**) Volcano plots of DEGs in SNI-2w (**b**) and SNI-4w (**c**) group compared to sham group. The red and blue dots indicate significantly up-regulated and down-regulated genes, respectively. (**d**,**e**) Hierarchical cluster analysis of SNI-2w (**d**) and SNI-4w (**e**) group compared to sham group. The dendrograms represent the classification of genes. The number in the color scale indicates the Log2 fold change values (Log2FC). The heatmap shows the top 20 genes (red) with the greatest positive Log2FC and the bottom 20 genes with the greatest negative (blue) Log2FC.

**Figure 3:**
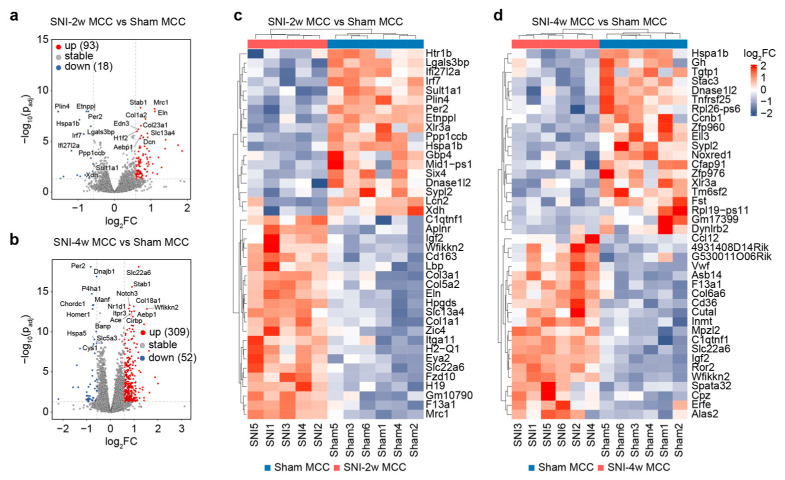
General transcriptomic outcomes of MCC. (**a**,**b**) Volcano plots of DEGs in SNI-2w (**a**) and SNI-4w (**b**) group compared to sham group. The red and blue dots indicate significantly up-regulated and down-regulated genes, respectively. (**c**,**d**) Hierarchical cluster analysis of SNI-2w (**c**) and SNI-4w (**d**) group compared to sham group. The dendrograms represent the classification of genes. The number in the color scale indicates the Log2 fold change values (Log2FC). The heatmap shows the top 20 genes (red) with the greatest positive Log2FC and the bottom 20 genes with the greatest negative (blue) Log2FC.

We next examined the expression profiles of DEGs in cingulate subregions following SNI. Transcriptome-wide sequencing detected approximately 37,536 and 37,861 expressed genes in the ACC and MCC, respectively, at two weeks post-SNI, followed by 38,103 and 38,985 genes at four weeks. PCA revealed a tendency toward separation among the six experimental groups, indicating distinct transcriptional patterns among the groups (Figure 2a). The number of altered genes increased with longer post-injury intervals. In the ACC, volcano plot analysis identified 63 up- and 62 down-regulated genes at 2 weeks post-SNI, and 195 up- and 384 down-regulated genes at 4 weeks, compared with sham controls (Figure 2b,c). Correspondingly, in the MCC we detected 93 up- and 18 down-regulated genes at 2 weeks, and 309 up- and 52 down-regulated genes at 4 weeks (Figure 3a,b). A heatmap of the top 40 DEGs (ranked by fold change) further confirmed clear segregation between experimental and control groups (Figure 2d,e and Figure 3c,d). Across both regions and time points, protein-coding RNAs accounted for most transcriptional changes (81.8–94.4%), followed by long non-coding RNAs (4.7–10.8%) and pseudogenes (0.9–6.5%) (Figure A2d).

**Figure 4:**
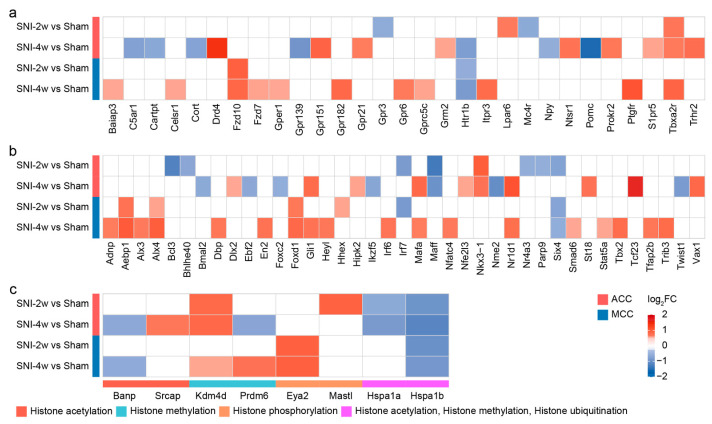
Heatmaps of the representative DEGs in the cingulate cortex after nerve injury. (**a**–**c**) DEGs of synapse-related molecules, G protein-coupled receptors, ion channels, neuropeptides (**a**), transcription factors (**b**), and epigenetic regulators (**c**) in the cingulate cortex after nerve injury. Colors in the heatmaps indicate the Log2FC values among the different datasets. The up- and down-regulated genes are colored in red and blue, respectively. Under panel c, the bottom-row annotation designates the functional categories of epigenetic modifiers. The heatmap was generated by intersecting all DEGs with genes from Table A2 and datasets of known transcriptional factors and epigenetic regulators obtained from public sources (Refs. [59,68]).

Synapse-related molecules, G protein-coupled receptors (GPCRs), ion channels, and neuropeptides play critical roles in the transmission and modulation of nociceptive signals [43,44,45]. Based on prior annotations [46,47], we curated a reference set of genes (Table A2) within these functional categories and cross-referenced them with our DEGs (Supplemental Data S1). This analysis revealed a notable enrichment of DEGs encoding GPCRs, followed by neuropeptides, and a few synapse-associated molecules, whereas no significant changes were observed in ion channels (Figure 4a). Among the altered neuropeptides, we detected differential expression of *Npy*—which is known to modulate pain at both spinal and supraspinal levels [48,49]—and *Prok2*, previously implicated in NP and negative affect via the nucleus accumbens [50]. Within the GPCR category, several receptors with established roles in pain processing showed altered expression, including the class A GPCRs *Mc4r* [51] and *Htr1b* [52], the G protein-coupled estrogen receptor *Gper1* [53], and the class C metabotropic glutamate receptor *Grm2* [54]. Although not previously linked directly to pain, *Baiap3*—a gene implicated in dense-core vesicle trafficking and post-synaptic neurotransmission deficits—has been shown to regulate depressive-like behaviors in the prefrontal cortex [55].

Emerging evidence underscores the role of transcription factors (TFs) in chronic pain [56,57,58]. From a public source [59], we identified a broader repertoire of TFs with altered expression in the cingulate cortex under NP conditions (Figure 4b). These include: *Bcl3*, an atypical IκB family protein that modulates NF-κB activity [60]; *Irf7*, a member of the interferon regulatory factor family [61]; *Hipk2*, an evolutionarily conserved serine/threonine kinase involved in neuronal development and homeostasis [62]; and *Tfap2b*, an AP-2 family transcription factor implicated in organ development and differentiation [63]. All four have been linked to pain regulation in the spinal cord via neuroinflammatory mechanisms [61,64,65,66]. Notably, *Bcl3* and *Irf7* were down-regulated in the NP model, whereas *Hipk2* and *Tfap2b* were up-regulated. Additionally, we observed upregulation of *Adnp*, a key factor in brain formation and function. Although not previously associated directly with pain, ADNP has been shown to modulate synaptic plasticity in ACC neurons through Wnt signaling and to counteract anesthesia-induced social deficits [67].

Broad transcriptional changes are often associated with extensive chromatin remodeling and epigenetic regulation. Among the 720 known epigenetic regulators [68], we observed altered expression of a limited set of genes implicated in histone modification when comparing sham and SNI mice across cingulate subregions (Figure 4c). These include *Banp* and *Srcap*, involved in histone acetylation; *Kdm4d* and *Prdm6*, associated with histone methylation; *Eya2* and *Mastl*, linked to histone phosphorylation; as well as *Hspa1a* and *Hspa1b*, which participate in histone acetylation, methylation, and ubiquitination. Notably, both *Hspa1a* and *Hspa1b*—which encode members of the heat shock protein family A—participate in pain modulation. While Hspa1a counteracts inflammatory pain by suppressing macrophage glycolysis, Hspa1b may promote NP through endoplasmic reticulum stress-related mechanisms at the level of the spinal dorsal horn (SDH) [69,70].

### 3.3. Functional Enrichment Analysis of the Differentially Expressed Genes After SNI

**Figure 5:**
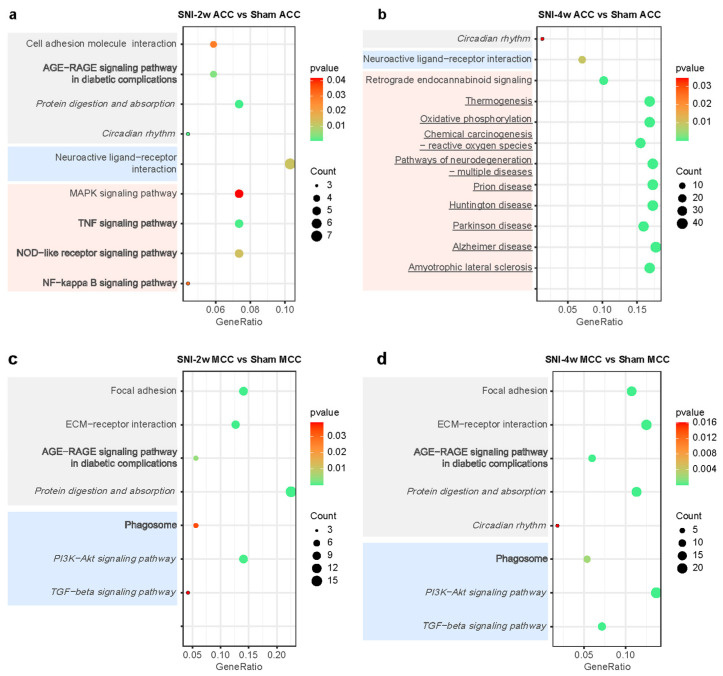
KEGG enrichment of DEGs in the cingulate cortex after SNI. (**a**–**d**) Bar plots for ACC (SNI 2w vs. Sham), ACC (SNI 4w vs. Sham), MCC (SNI 2w vs. Sham) and MCC (SNI 4w vs. Sham), respectively. Plain, bold, underlined, and italic text denote pathways related to synaptic transmission/plasticity, neuroinflammation, oxidative stress or other processes, respectively. Grey, blue and pink boxes indicate pathways shared between ACC and MCC, shared between the two ACC time points, and unique to individual ACC time points, respectively. Bubble size represents the number of genes per pathway, and colour indicates the *p*-value.

To delineate the functional consequences of the DEGs, KEGG pathway enrichment analysis was performed using thresholds of *p* < 0.05 and a minimum gene count of 3 per pathway (Supplemental Data S2). Under these criteria, 20, 25, 22 and 33 pathways were significantly enriched in the ACC SNI 2w versus Sham, ACC SNI 4w versus Sham, MCC SNI 2w versus Sham, and MCC SNI 4w versus Sham comparisons, respectively. The principal results are summarised in Figure 5. Collectively, the enriched pathways spanned three broad functional domains: (1) neural signaling and synaptic plasticity, represented by neuroactive ligand–receptor interaction, mitogen-activated protein kinase (MAPK), extracellular matrix (ECM)–receptor interaction, and retrograde endocannabinoid signaling; (2) immune–inflammatory responses, represented by tumour necrosis factor (TNF), NOD-like receptor, and NF-κB signaling pathways; and (3) a core axis of oxidative stress and mitochondrial dysfunction, which forms the molecular basis of neurodegeneration. Other enriched pathways included phosphoinositide 3-kinase-protein kinase B (PI3K)–Akt signaling and circadian rhythm, among others.

From a subregional perspective of the cingulate cortex, several pathways related to circadian rhythm, protein digestion and cell adhesion were conserved at both time points in both regions, suggesting a conserved functional reprogramming across the cingulate cortex in NP. The ACC exhibited broader pathway alterations than the MCC as some pathways were unique to the ACC. For instance, neuroactive ligand–receptor interaction was enriched at both time points. Whereas MAPK, NOD-like receptor, and NF-κB pathways were enriched only at the early time point, pathways associated with oxidative stress, mitochondrial dysfunction, and neurodegeneration were enriched only at the late time point, indicating a progressive stress response unique to the ACC. These results suggest that SNI induces region-specific and time-dependent transcriptomic remodeling in the cingulate cortex and may provide clues for understanding the divergent molecular mechanisms of cingulate subregions in NP.

**Figure 6:**
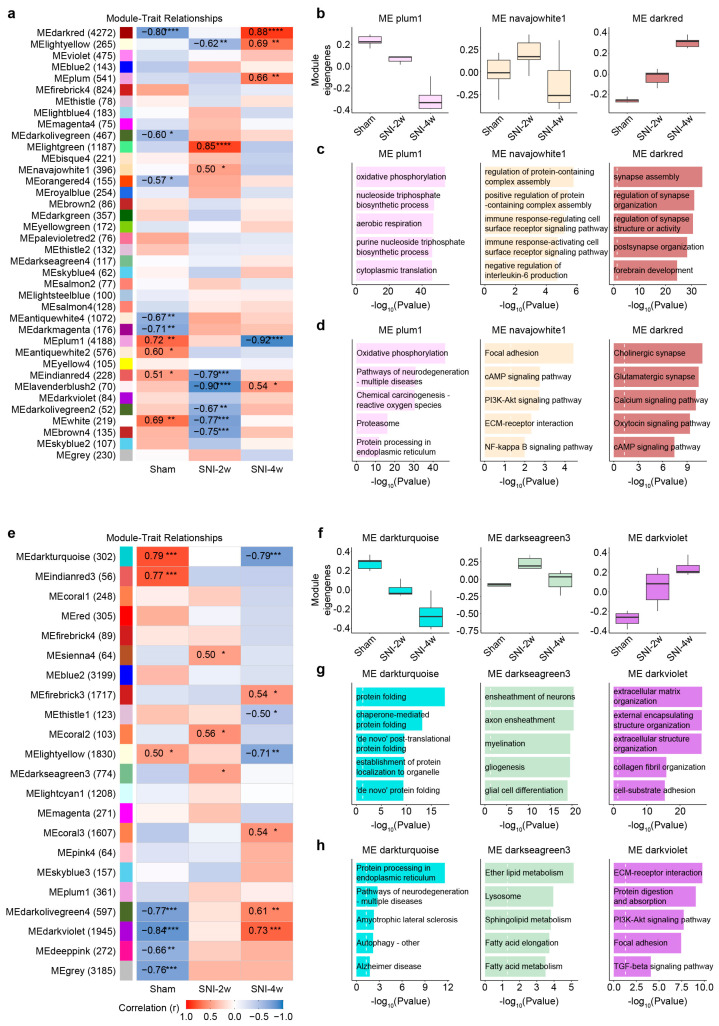
WGCNA. (**a**) Correlation analysis between gene modules and phenotypes in the ACC. The heatmap presents correlation coefficients and associated p-values between each gene co-expression module and the phenotypic groups (sham, SNI-2w, and SNI-4w). Positive (r > 0) and negative (r < 0) correlations indicate, respectively, up- or down-regulated co-expression patterns for each module in a given group, and color intensity denotes the strength of the correlation. * indicates *p* value < 0.05 for NP trait, ** indicates *p* value < 0.01, *** indicates *p* value < 0.001, and **** indicates *p* value < 0.0001. (**b**) Boxplots showing the distribution of module expression (mean log_2_(CPM + 1) of all genes within a given module) for different groups. (**c**,**d**) Enrichment analysis of the three selected module. Representative enriched biological process terms and KEGG pathways for each module are displayed in (**c**,**d**), respectively. The left panels correspond to the sham group, the middle panels to the SNI-2w group, and the right panels to the SNI-4w group. (**e**–**h**) WGCNA network in the MCC. The conventions are the same as those in (**a**–**d**).

**Figure A3:**
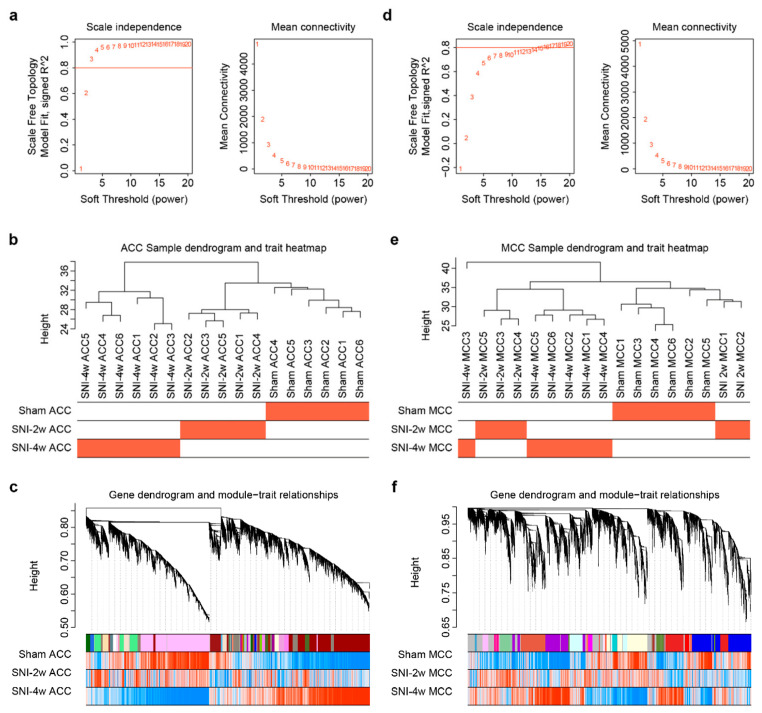
Construction of WGCNA network. (**a**) Weighting coefficient β selection in the ACC. The left panel displays the scale-free topology model fit index (R^2^) across a range of soft-thresholding powers, while the right panel shows the corresponding average connectivity. Connectivity distribution of nodes with β set as 3 (**left panel**) and the verification of scale-free topology with β set as 3 (**right panel**). (**b**) Modules were clustered, and correlations were investigated with a sample dendrogram. (**c**) Gene clustering dendrogram and module detection. The dendrogram illustrates hierarchical clustering of genes, with distinct modules identified using the dynamic tree-cutting method and indicated by assigned colors. (**d**–**f**) Construction of WGCNA network in the MCC. Connectivity distribution of nodes with β set as 14 (**left panel** in **d**) and the verification of scale-free topology with β set as 14 (**right panel** in **d**). The conventions are the same as those in (**a**–**c**).

To complement our KEGG analysis of DEGs, we next constructed WGCNA networks for both subregions (Figure 6 and Figure A3). A scale-free topology fit (R^2^ > 0.8) was selected. Modules were identified using topological overlap matrices with a dynamic tree-cutting algorithm, setting a minimum module size of 50 genes. Modules with high similarity (cut height = 0.3) were merged, yielding 38 distinct co-expression modules in the ACC (module size range: 52 genes in ‘darkolivegreen’ to 4272 genes in ‘darkred’; Figure 6a) and 22 modules in the MCC (module size range: 56 genes in ‘indianred3’ to 3199 genes in ‘blue2’; Figure 6e). To explore the biological relevance of ACC-associated modules, we correlated module eigengenes with experimental groups. Several modules showed significant associations with NP states (Figure 6a,b). The plum1 module was positively correlated with the sham group (r = 0.72, *p* < 0.01), whereas navajowhite1 correlated with the SNI-2w group (r = 0.50, *p* < 0.05). The darkred module showed a strong positive correlation with the SNI-4w group (r = 0.88, *p* < 0.0001). These three modules were therefore selected for further enrichment analysis. KEGG and Gene Ontology biological process analyses revealed that plum1 was enriched for pathways related to oxidative phosphorylation, including Proteasome, Protein processing in endoplasmic reticulum, and Pathways of neurodegeneration—multiple diseases; Navajowhite1 was enriched in immune-response pathways such as ECM–receptor interaction, PI3K–Akt signaling, and NF-κB signaling; Darkred was associated with synaptic structure and function, including glutamatergic synapse, calcium signaling, and cyclic adenosine monophosphate (cAMP) signaling pathways (Figure 6c,d).

Similarly, in the MCC, three key modules were identified based on significant correlations with experimental groups (Figure 6e,f). The darkturquoise module was positively correlated with the sham group (r = 0.79, *p* < 0.001), darkseagreen3 with SNI-2w (r = 0.49, *p* < 0.05), and darkviolet with SNI-4w (r = 0.73, *p* < 0.001). Enrichment analysis showed that darkturquoise was associated with protein-folding pathways including Autophagy, Protein processing in endoplasmic reticulum, and Pathways of neurodegeneration—multiple diseases; Darkseagreen3 was enriched in axon ensheathment-related terms such as ether lipid metabolism, sphingolipid metabolism, and Fatty acid metabolism; Darkviolet was linked to ECM organization, involving PI3K–Akt signaling, TGF-β signaling, and ECM–receptor interaction (Figure 6g,h). Collectively, the WGCNA results substantiate the preceding KEGG pathway analysis, underscoring the pivotal roles of synaptic transmission/plasticity and neuroinflammatory mechanisms in the pathophysiology of NP. Furthermore, these findings suggest the possible involvement of proteostasis disruption and mitochondrial dysfunction—pathological mechanisms also seen in various neurodegenerative disorders—in NP pathogenesis.

### 3.4. Protein–Protein Interaction Analysis After SNI

**Figure 7:**
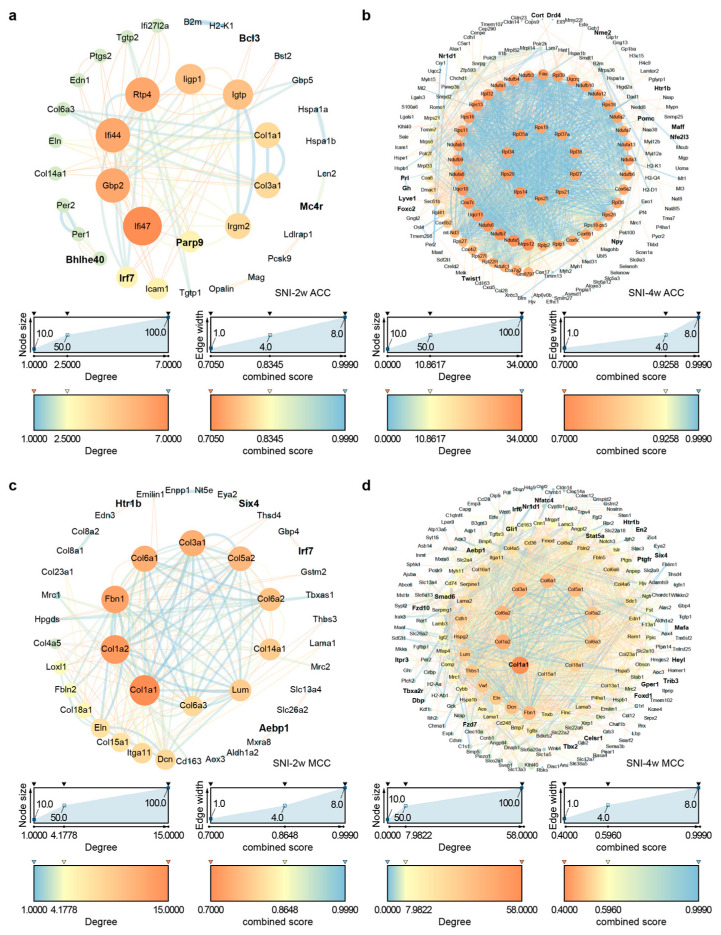
PPI networks of the DEGs in SNI groups comparing the sham group. (**a**,**b**) STRING PPI networks illustrating potential interactions among DEGs in the ACC at 2 weeks (**a**) and 4 weeks (**b**) post-SNI. (**c**,**d**) STRING PPI networks illustrating potential interactions among DEGs in the MCC at 2 weeks (**c**) and 4 weeks (**d**) post-SNI. Node color and size correspond to the degree of connectivity; edge color and thickness correspond to the combined interaction score. Central nodes (hub genes) represent the top 10 DEGs ranked by connectivity degree, while peripheral nodes denote other DEGs with predicted interactions. Bold labels in the panels indicate genes categorized as synapse-related molecules, G protein-coupled receptors, ion channels, neuropeptides, or transcription factors.

To investigate the molecular interactions and identify potential key drivers among the DEGs in the ACC and MCC following SNI, we conducted PPI network analysis using the STRING database. In the ACC, the DEG network comprised 36 nodes with 90 edges in the SNI-2w group and 188 nodes with 2042 edges in the SNI-4w group. Among the top 10 hub genes in the SNI-2w group, the majority were interferon-stimulated genes encoding GTPase-active proteins (e.g., *Ifi47*, *Gbp2*, *Ifi44*, *Iigp1*, *Igtp*) along with core structural components of the ECM (*Col1a1* and *Col3a1*). In contrast, the top 10 hub genes in the SNI-4w group were exclusively ribosomal protein genes (Figure 7a,b). While recent studies have implicated interferon signaling in ACC-mediated neuroinflammation [71,72] and ECM remodeling in synaptic plasticity [73,74] during chronic pain, the functional role of ribosomal proteins in pain pathogenesis remains largely unexplored. Similarly, in the MCC, network analysis revealed 45 nodes with 188 edges in the SNI-2w group and 225 nodes with 1796 edges in the SNI-4w group. Notably, the top 10 hub DEGs in both the 2-week and 4-week MCC networks were predominantly essential constituents of the ECM (Figure 7c,d), again highlighting dysregulation of cingulate ECM organization underlying the pathological progression of NP.

### 3.5. Shared Transcriptional Changes in Cingulate Subregions Across Time Points

**Figure 8:**
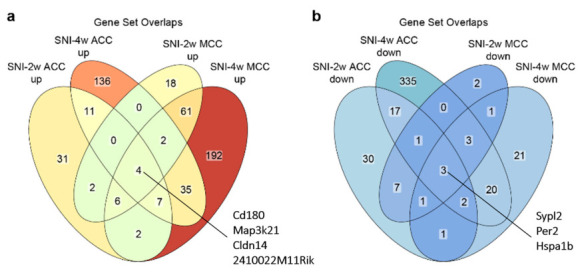
Integrated analysis of transcriptomic data. (**a**,**b**) Venn diagram depicting partial overlaps of up-regulated (**a**) and down-regulated (**b**) DEGs of SNI-2w and SNI-4w groups compared to sham group in cingulate subregions.

By Venn diagram analysis, we identified seven overlapping targets (Figure 8). Among these, four genes—*Cd180* (encoding CD180 antigen), *Map3k21* (encoding mitogen-activated protein kinase kinase kinase 21), *Cldn14* (encoding claudin-14), and *2410022M11Rik* (a functionally uncharacterized non-coding RNA)—were consistently up-regulated across different post-injury time points and cingulate subregions following SNI. Conversely, three genes—*Sypl2* (encoding synaptophysin-like 2), *Per2* (encoding period circadian regulator 2), and *Hspa1b* (encoding heat shock protein family A member 1B)—showed consistent down-regulation under the same conditions. In prior KEGG pathway analysis, *Per2* was enriched in the circadian rhythm pathway, while *Hspa1b* and *Map3k21* were enriched in the MAPK signaling pathway (Figure 5). Notably, Per2 has been demonstrated to contribute to NP via neuroinflammatory mechanisms within the SDH [75].

## 4. Discussion

Prior cingulate transcriptomic studies in NP were largely limited to early time points or the ACC alone. Our study addresses this gap by systematically profiling temporal transcriptomic dynamics across two subregions of the cingulate cortex following prolonged peripheral nerve injury. We observed that the number of DEGs and significantly enriched KEGG pathways increased over time within each cingulate subregion. Functional analysis not only corroborates previously established roles for synaptic transmission/plasticity and neuroinflammation in NP, but also offers a potentially novel perspective: at the cortical level, NP may involve transcriptomic alterations in mitochondrial function, which are also implicated in neurodegenerative disorders.

### 4.1. Cingulate Synaptic Mechanisms in Neuropathic Pain

Chronic pain is sustained by central sensitization—a form of synaptic plasticity characterized by heightened neuronal responsiveness within central nociceptive pathways following injury. LTP of glutamatergic transmission in the ACC represents a well-established model for studying the synaptic mechanisms underlying chronic pain [7]. Our transcriptomic profiling revealed an enrichment of DEGs in synaptic-related functions and pathways in the cingulate cortex of NP mice, consistent with prior transcriptomic studies of the ACC [21,22,23,24]. We now proceed to review the cingulate synaptic mechanisms under NP conditions based on the pathways we have enriched.

Peripheral nerve injury elicits excessive glutamate release, promoting Ca^2+^ influx into postsynaptic neurons through the activation of N-Methyl-D-Aspartate Receptors (NMDARs). The expression of NP-related cingulate LTP involves a multi-step signaling cascade dependent on Ca^2+^-calmodulin. Central to this cascade, adenylyl cyclase 1 converts ATP to cAMP, which in turn activates cAMP-dependent protein kinase A (PKA). PKA promotes the synthesis of diverse plasticity-related proteins via phosphorylation of the transcription factor cAMP response element-binding protein (CREB) [7,20]. In addition to cAMP signaling, activation of the MAPK pathway also contributes to CREB phosphorylation [76]. Collectively, these biochemical events enhance the phosphorylation and membrane trafficking of NMDARs and Ca^2+^-permeable AMPARs, thereby amplifying postsynaptic responsiveness [7,20]. Correspondingly, selective pharmacological inhibition of the cAMP [77] or MAPK [78,79] pathway in the ACC attenuates behavioral hypersensitivity and associated negative affect in preclinical models of chronic pain. In addition to intracellular signaling pathways, the ECM is also recognized for its influence on synaptic plasticity and its participation in diverse pathophysiological processes in the central nervous system [80]. Supporting this, recent studies demonstrate that a key ECM component laminin-1 mediates the transduction of extracellular changes into intracellular synaptic remodeling in the ACC, thereby contributing to the development of NP and associated negative emotions [73,74]. Our data demonstrate an enrichment of KEGG pathways central to synaptic modulation—including ECM, glutamatergic synapse, calcium, cAMP, and MAPK signaling—thereby supporting cingulate synaptic dysregulation in NP.

### 4.2. Cingulate Neuroinflammation Mechanisms in Neuropathic Pain

Central sensitization is also driven by neuroinflammation within both the peripheral and central nervous systems. A hallmark of neuroinflammation is the activation of glial cells—including microglia and astrocytes—in the spinal cord and brain, which promotes the release of proinflammatory cytokines and chemokines [81]. Under chronic pain conditions, astrocyte activation occurs in the ACC [82], leading to elevated levels of inflammatory cytokines such as TNF-α. TNF-α enhances synaptic transmission in the ACC via presynaptic mechanisms and also promotes necroptosis of parvalbumin-expressing interneurons, collectively shifting the excitatory–inhibitory balance toward excitation [83,84]. Accordingly, intra-ACC administration of anti-TNF-α antibodies alleviates behavioral hypersensitivity and negative emotions associated with chronic pain [83,85,86]. Furthermore, NF-κB expression is markedly increased in the ACC under chronic pain conditions [87], and its selective inhibition attenuates pain-related hypersensitivity and affective disturbances [88]. Mechanistically, elevated TNF-α in the ACC activates the NF-κB pathway, up-regulating acid-sensing ion channel 1a and subsequently enhancing the activity of ACC glutamatergic neurons [88]. Previous transcriptomic studies have confirmed that DEGs in the ACC under chronic pain conditions are enriched in neuroinflammation-related pathways [22,25]. In line with these findings, our study reveals that DEGs in the ACC under NP are enriched in TNF and NF-κB signaling pathways, again underscoring cortical neuroinflammatory mechanisms in the pathogenesis of chronic pain.

### 4.3. Cingulate Mitochondrial Mechanisms in Neuropathic Pain

Beyond the well-established mechanisms of synaptic plasticity and neuroinflammation central to current research on ACC-mediated chronic pain, our functional analyses also reveal previously understudied regulatory pathways. Chronic pain shares potential mechanistic links with neurodegenerative diseases such as Alzheimer’s disease and amyotrophic lateral sclerosis. Sustained pain can induce a persistent neuroinflammatory state, disrupt proteostasis [89], and cause mitochondrial dysfunction [90,91], processes analogous to those observed in neurodegeneration [92]. Having already addressed cingulate neuroinflammation mechanisms in NP, we now review mitochondrial mechanisms.

The “mitotoxicity hypothesis”, first proposed in the context of chemotherapy-induced neuropathy, has subsequently been validated in models of peripheral nerve injury. These insults induce mitochondrial dysfunction in primary sensory neurons by disrupting key mitochondrial processes (including bioenergetics, transport, fusion, and mitophagy) and elevating nitro-oxidative stress. Together, these alterations impair axonal growth and promote neuronal sensitization [93,94]. Mitophagy represents a central mitochondrial quality-control mechanism. Its dysregulation in the peripheral nervous system leads to the accumulation of defective mitochondria, increased reactive oxygen species (ROS) generation, microglial activation, and myelin breakdown, thereby contributing to NP development [95,96]. At the SDH level, deficient mitophagy in microglia facilitates NOD-, LRR- and pyrin domain-containing protein 3 (NLRP3) inflammasome-driven neuroinflammation via mitochondrial ROS, ultimately promoting pain-related plasticity [97]. Accordingly, interventions that enhance mitophagy demonstrated therapeutic efficacy in experimental NP models [93,97]. Notably, recent work has revealed that the PINK1/PARKIN-dependent mitophagy pathway is impaired in the ACC of NP rats. Treadmill exercise was shown to restore mitochondrial homeostasis by reactivating this pathway, leading to alleviation of pain-like behaviours [98]. These findings, together with our analyses, suggest a potential involvement of mitochondrial dysfunction in the ACC as an underexplored component of NP pathophysiology; however, functional validation is required to confirm this possibility.

### 4.4. Other Potential Pathways of Neuropathic Pain in the Cingulate Cortex

Typically activated by cytokines and growth factors, the PI3K/Akt signalling orchestrates the activation or suppression of a broad spectrum of downstream effectors involved in cell survival, proliferation, and metabolism [99]. To our knowledge, only a single study has reported that inhibiting PI3K/Akt signalling in the ACC ameliorates both nociceptive responses and affective behaviors in a rodent model of cancer pain [100], highlighting a significant gap in our understanding of its role in NP at cortical levels. Circadian modulation of NP is well-documented clinically [101], but mechanistic studies have largely focused on peripheral and spinal sites, along with descending pain modulatory systems [102]. Direct evidence for the involvement of higher-order cortical and limbic structures in circadian gating of pain remains scarce. Nevertheless, molecular oscillations with clear diurnal rhythms have been observed within these regions [103]. Transcriptomic studies have revealed significant dysregulation of core clock gene expression in the ACC under major depressive disorder [104]. Our findings similarly link circadian disruption in the cingulate cortex to NP pathogenesis. Thus, future studies should elucidate how cortical circuits contribute to the circadian organization of pain processing.

### 4.5. Limitations

First, it is well established that there are significant sex differences in the pathogenesis, clinical manifestations, and molecular mechanisms of NP [105]. Recently, Dai et al. demonstrated sex-dependent transcriptional and pathway alterations in the ACC following nerve injury [21]. Thus, the exclusive use of male mice may have missed sex-specific molecular mechanisms. Second, the cingulate cortex comprises heterogeneous cell populations, including distinct neuronal and glial subtypes. All bioinformatic analyses reported here were derived from bulk tissue, which integrates signals across these diverse cell types. Under NP conditions, glial cells can increase in proportion [81]. Such compositional changes alone could give rise to differential gene expression signals in bulk RNA-seq, independently of transcriptional alterations within a given cell type. Future work employing single-cell RNA transcriptomics will be essential to dissect cell-type proportion shifts and cell-type-specific transcriptional changes following NP. Third, while our study focused on transcriptional and pathway-level alterations, it did not assess post-transcriptional molecular biological processes. Integrating multi-omics approaches, such as proteomics and metabolomics, could reveal more refined molecular distinctions underlying NP. Fourth, whether the identified transcriptomic changes causally contribute to the initiation or persistence of NP, and whether they represent viable therapeutic targets, remains to be experimentally validated in further mechanistic and interventional studies. Fifth, Majumdar et al. recently demonstrated how peripheral nerve injury induces coordinated proteomic changes across the sciatic nerve, spinal cord, and orbitofrontal cortex [106]. Given that both the orbitofrontal and cingulate cortex are involved in affective and cognitive aspects of pain [107], future multi-regional transcriptomic or proteomic studies [21,24,106] spanning the prefrontal cortex will be essential to determine the shared and region-specific molecular signatures that drive the affective dimension of chronic pain.

## 5. Conclusions

Collectively, these findings substantiate the involvement of synapse- and neuroinflammation-related mechanisms within the cingulate cortex in the pathophysiology of NP. Furthermore, our transcriptomic data point to a potential involvement of mitochondrial dysfunction in the cingulate cortex, although functional validation is required. Our data contribute to a deeper understanding of NP etiology and may offer a theoretical foundation for developing novel therapeutic strategies.

## Data Availability

All raw sequence data have been deposited in the NCBI Sequence Read Archive under the accession number PRJNA1401302 (available at: https://dataview.ncbi.nlm.nih.gov/object/PRJNA1401302, accessed on 26 June 2026).

## Supplementary Materials

The following supporting information can be downloaded at: https://www.mdpi.com/article/10.3390/biomedicines14071495/s1. Supplemental Data S1: Altered transcriptional signatures in the cingulate cortex after SNI related to Figure 4. Supplemental Data S2: KEGG analysis of DEGs of the subregions of the cingulate cortex at 2 and 4 weeks post-SNI.

## Abbreviations

The following abbreviations are used in this manuscript:

ACC	Anterior Cingulate Cortex
cAMP	Cyclic Adenosine Monophosphate
CREB	cAMP Response Element-Binding Protein
DEGs	Differentially Expressed Genes
ECM	Extracellular Matrix
GO	Gene Ontology
GPCRs	G Protein-Coupled Receptors
KEGG	Kyoto Encyclopedia of Genes and Genomes
LTP	Long-Term Potentiation
MAPK	Mitogen-Activated Protein Kinase
MCC	Midcingulate Cortex
NF-κB	Nuclear Factor Kappa B
NLRP3	NOD-, LRR- and Pyrin Domain-Containing Protein 3
NMDAR	N-Methyl-D-Aspartate Receptor
NP	Neuropathic Pain
PI3K-Akt	Phosphoinositide 3-Kinase–Protein Kinase B
PKA	Protein Kinase A
PPI	Protein–Protein Interaction
ROS	Reactive Oxygen Species
SDH	Spinal Dorsal Horn
SNI	Spared Nerve Injury
TFs	Transcription Factors
TGF-β	Transforming Growth Factor-Beta
TNF	Tumor Necrosis Factor
WGCNA	Weighted Gene Co-expression Network Analysis

## Appendix A

**Table A1:** The summary of raw general RNA-sequencing dataset in RNA sequencing.

Sample	Raw Data Read	Valid Data Read	Valid Ratio %	Mapped Rate %	Q20%	Q30%
Sham ACC1	21818051	21643187	99.20	98.70	99.03	95.66
Sham ACC2	21587870	21420444	99.22	98.70	98.99	95.40
Sham ACC3	21569341	21398228	99.21	98.80	99.02	95.53
Sham ACC4	21947667	21775563	99.22	98.80	99.01	95.50
Sham ACC5	21869294	21700825	99.23	98.70	99.01	95.49
Sham ACC6	22152128	21975089	99.20	98.80	99.01	95.52
SNI-2w ACC1	22308621	22121100	99.16	98.80	99.00	95.48
SNI-2w ACC2	23851517	23652719	99.17	98.80	98.96	95.27
SNI-2w ACC3	26272018	26032632	99.09	98.80	98.97	95.33
SNI-2w ACC4	26117787	25882816	99.10	98.80	98.93	95.17
SNI-2w ACC5	25422486	25222963	99.22	98.70	98.99	95.41
SNI-4w ACC1	24424491	24231291	99.21	98.70	98.95	95.28
SNI-4w ACC2	26116098	25886414	99.12	98.50	98.94	95.24
SNI-4w ACC3	24611453	24409985	99.18	98.70	98.96	95.28
SNI-4w ACC4	23637283	23454763	99.23	98.80	98.94	95.15
SNI-4w ACC5	25872247	25683668	99.27	98.80	99.00	95.42
SNI-4w ACC6	25735743	25524141	99.18	98.80	99.00	95.48
Sham MCC1	28310668	28075670	99.17	98.70	98.98	95.37
Sham MCC2	26206389	25981769	99.14	98.70	98.99	95.41
Sham MCC3	24960891	24764247	99.21	98.60	98.96	95.25
Sham MCC4	24349730	24140681	99.14	98.60	98.97	95.33
Sham MCC5	24049610	23868263	99.25	99.00	99.08	95.79
SNI-2w MCC1	26830426	26639099	99.29	98.90	99.06	95.67
SNI-2w MCC2	25049360	24860351	99.25	98.90	99.03	95.54
SNI-2w MCC3	25136432	24939615	99.22	98.80	98.97	95.35
SNI-2w MCC4	24317017	24159519	99.35	98.90	99.07	95.67
SNI-2w MCC5	25713271	25505292	99.19	98.80	98.94	95.21
SNI-2w MCC6	24828567	24621943	99.17	98.80	99.00	95.42
SNI-4w MCC1	27693727	27465768	99.18	98.70	98.94	95.20
SNI-4w MCC2	26213380	26003592	99.20	98.8	98.97	95.29
SNI-4w MCC3	25283618	25087761	99.23	98.70	98.99	95.42
SNI-4w MCC4	29172720	28923281	99.14	98.70	98.97	95.37
SNI-4w MCC5	21818051	21643187	99.20	98.70	99.03	95.66
SNI-4w MCC6	21587870	21420444	99.22	98.70	98.99	95.40

**Table A2:** A reference set of genes encoding synapse-related molecules, G protein-coupled receptors, ion channels, and neuropeptides.

Types	Genes
Synapse-related molecules	Agt Cadps2 Chat Cplx1 Napb Slc17a7 Slc18a3 Slc5a7 Snca Sncg Stxbp5l Sv2b Syt6 Syt9 Ywhaz Abat Atp2a2 Atp2b2 Baiap3 Cadps Camk2a Cd47 Clu Lin7a Nat8l Nlgn1 Nrxn3 Ntrk2 Pak1 Pde1b Rph3a Sirpa Slc17a6 Slc1a2 Slc6a1 Slc6a7 Sv2a Syn2 Syp Syt11 Vamp1 Ryr2 Ryr3 Itpr1 Itpr2 Itpr3 Nlgn1 Pde4b Calm1
Ion channels	Ano1 Cacnb3 Cacng5 Chrna3 Chrna4 Chrnb3 Chrnb4 Kcna2 Kcnd2 Kcng4 Kcnip1 Kcnip3 Kcnma1 Kcnmb4 Lrrc55 Cacna1b Cacna1g Cacna1i Cacna2d1 Cacng2 Gabrb1 Gabrg2 Gabrg3 Glrb Hcn2 Kcna6 Kcnab2 Kcnc1 Kcnc2 Kcnc4 Kcnip4 Kcnj3 Scn1a Scn1b Ttyh1 Gabra1 Gabra2 Gabra3 Gabra4 Gabra5 Gabrb1 Gabrb2 Gabrb3 Gria1 Gria2 Gria3 Gria4 Grid1 Grid2 Grik1 Grik2 Grik3 Grik4 Grik5 Grin1 Grin2a Grin2b Grin2c Grin3a P2rx1 P2rx4 P2rx7
G protein-coupled receptors	Ackr1 Ackr3 Adcyap1r1 Adgra1 Adgra2 Adgra3 Adgrb1 Adgrb2 Adgrb3 Adgrd1 Adgre1 Adgre5 Adgrf4 Adgrf5 Adgrg1 Adgrg2 Adgrg3 Adgrg6 Adgrl1 Adgrl2 Adgrl3 Adgrl4 Adgrv1 Adora1 Adora2b Adora3 Adra1a Adra1b Adra2a Adra2b Adra2c Adrb1 Adrb2 Adrb3 Agtr2 Agtrap ApInr Avpr1a C3ar1 C5ar1 C5ar2 Calcrl Cckbr Ccr1 Ccr10 Ccr5 Ccr7 Ccrl2 Celsr1 Celsr2 Celsr3 Chrm1 Chrm2 Chrm3 Chrm5 Cmklr1 Cnr1 Crcp Crhr1 Crhr2 Cx3cr1 Cxcr2 Cxcr4 Cysltr1 Drd1 Drd4 Drd5 Ednra Ednrb F2r F2rl3 Ffar1 Fpr2 Fzd1 Fzd10 Fzd2 Fzd3 Fzd4 Fzd5 Fzd6 Fzd7 Fzd8 Fzd9 Gabbr1 Gabbr2 Galr1 Ghsr Gipr Gper1 Gpr1 Gpr101 Gpr12 Gpr135 Gpr139 Gpr141 Gpr149 Gpr150 Gpr151 Gpr152 Gpr153 Gpr156 Gpr157 Gpr158 Gpr160 Gpr161 Gpr162 Gpr17 Gpr173 Gpr176 Gpr182 Gpr19 Gpr21 Gpr22 Gpr26 Gpr27 Gpr3 Gpr34 Gpr35 Gpr37 Gpr37l1 Gpr4 Gpr45 Gpr50 Gpr55 Gpr6 Gpr61 Gpr62 Gpr68 Gpr75 Gpr83 Gpr85 Gpr88 Gprc5b Gprc5c Grm1 Grm2 Grm3 Grm4 Grm5 Grm7 Grm8 Grpr Hcrtr1 Hcrtr2 Hrh1 Hrh2 Hrh3 Htr1a Htr1b Htr1d Htr1f Htr2a Htr2c Htr4 Htr5a Htr5b Htr6 Htr7 Kiss1r Lgr4 Lgr5 Lgr6 Lpar1 Lpar4 Lpar6 Ltb4r1 Mc1r Mc3r Mc4r Mchr1 Mtnr1a Nmbr Nmur2 Npbwr1 Npr3 Npy1r Npy2r Npy5r Ntsr1 Ntsr2 Ogfr Ogfrl1 Oprk1 Oprl1 Oprm1 Oxgr1 Oxtr P2ry1 P2ry12 P2ry13 P2ry14 P2ry2 P2ry6 Pgr15L Ppard Prokr2 Ptgdr Ptger1 Ptger2 Ptger3 Ptger4 Ptgfr Ptgir Pth1r Ramp1 Ramp2 Ramp3 Rorb Rxfp1 Rxfp2 Rxfp3 S1pr1 S1pr2 S1pr3 S1pr5 Sigmar1 Smo Sstr1 Sstr2 Sstr3 Sstr4 Sstr5 Sucnr1 Tacr1 Tacr3 Tas1r1 Tas1r3 Tbxa2r Trhr Trhr2 Uts2r Vipr1 Vipr2 Vmn1r4 Vmn2r29 Vmn2r84 Vmn2r87 Xcr1
Neuropeptides	Adcyap1 Agrp Calca Cartpt Cort Gal Grp Hcrt Nmb Nms Npb Npff Nppa Nps Npy Pcsk1n Pdyn Penk Pnoc Pomc Prok2 Pth2 Qrfp Scg5 Tac1 Tac2 Ecel1 Tenm1 Tyro3 Rapgef2 Sort1
